# Antiretroviral Therapy Program Expansion in Zambézia Province, Mozambique: Geospatial Mapping of Community-Based and Health Facility Data for Integrated Health Planning

**DOI:** 10.1371/journal.pone.0109653

**Published:** 2014-10-17

**Authors:** Troy D. Moon, Ezequiel B. Ossemane, Ann F. Green, Elisée Ndatimana, Eurico José, Charlotte P. Buehler, C. William Wester, Sten H. Vermund, Omo Olupona

**Affiliations:** 1 Vanderbilt Institute for Global Health, Nashville, Tennessee, United States of America; 2 Friends in Global Health, LLC, Maputo, Mozambique; 3 World Vision, Maputo, Mozambique; Brighton, Ethiopia

## Abstract

**Objective:**

To generate maps reflecting the intersection of community-based Voluntary Counseling and Testing (VCT) delivery points with facility-based HIV program demographic information collected at the district level in three districts (Ile, Maganja da Costa and Chinde) of Zambézia Province, Mozambique; in order to guide planning decisions about antiretroviral therapy (ART) program expansion.

**Methods:**

Program information was harvested from two separate open source databases maintained for community-based VCT and facility-based HIV care and treatment monitoring from October 2011 to September 2012. Maps were created using ArcGIS 10.1. Travel distance by foot within a 10 km radius is generally considered a tolerable distance in Mozambique for purposes of adherence and retention planning.

**Results:**

Community-based VCT activities in each of three districts were clustered within geographic proximity to clinics providing ART, within communities with easier transportation access, and/or near the homes of VCT volunteers. Community HIV testing results yielded HIV seropositivity rates in some regions that were incongruent with the Ministry of Health’s estimates for the entire district (2–13% vs. 2% in Ile, 2–54% vs. 11.5% in Maganja da Costa, and 23–43% vs. 14.4% in Chinde). All 3 districts revealed gaps in regional disbursement of community-based VCT activities as well as access to clinics offering ART.

**Conclusions:**

Use of geospatial mapping in the context of program planning and monitoring allowed for characterizing the location and size of each district’s HIV population. In extremely resource limited and logistically challenging settings, maps are valuable tools for informing evidence-based decisions in planning program expansion, including ART.

## Introduction

In recent years, the world’s governments have galvanized unprecedented support towards the scale-up of HIV care and treatment. International programs such as the President’s Emergency Plan for AIDS Relief (PEPFAR) and the Global Fund to Fight AIDS, Tuberculosis and Malaria have provided funding and technical assistance for the successful initiation of large numbers of patients on antiretroviral therapy (ART) in sub-Saharan Africa and elsewhere. [1]–[4] The absolute number of persons with access to and initiating ART is unprecedented (>10 million as of late 2013), yet key challenges remain to be addressed as to the quality of services. Gaps include suboptimal awareness of personal HIV status, late enrollment into HIV care and treatment, poor adherence and retention strategies, minimal psychosocial support initiatives, limited diagnostics of opportunistic infections, depleted human resources for health, inadequate integration of HIV services, and needs for health system strengthening. [1], [4]–[10] Each of these requires thoughtful, efficient, and evidence-based health planning in order to have the desired impact.

Mozambique ranked 185 of 187 nations on the 2012 Human Development Index of the United Nations Development Program and was one of the original 15 PEPFAR focus countries. [11], [12] An estimated 2009 national adult HIV prevalence of 11.5% (∼1.4 million HIV-infected persons aged 15–49) ranked it among the most heavily HIV-afflicted nations in the world. [13] The magnitude of this epidemic is especially evident in Zambézia Province, Mozambique’s second largest province and home to 3.8 million persons. [5], [6], [13] While Mozambique ranks among the poorest of the poor nations, Zambézia Province consistently ranks among Mozambique’s bottommost performing provinces with low literacy rates, poor maternal and child health (MCH) indices, and high rates of tuberculosis, malaria, and malnutrition. [14]–[18] More HIV-infected persons (∼275,000) live in Zambézia than in any other province, representing 20% of Mozambique’s HIV-infected adolescents/adults as of 2009 [13], [19].

Estimating ART coverage rates for program planning purposes has been difficult, mainly due to weaknesses in HIV surveillance systems and in calculating HIV prevalence at a local site level. From 1988 to 2009, Mozambique relied on bi-annual serosurveys conducted at sentinel antenatal care (ANC) clinics (limited to 3–4 PMTCT clinics per province) for HIV prevalence estimates. [20] In 2009, Mozambique implemented its first and only population based National HIV epidemiologic survey, to date. [13] While confidence in these methods of determining HIV prevalence has increased, they continue to be limited to providing estimates for national and regional levels only, and do not provide estimates for the district or site level. Recently, there has been increasing interest in the use of routine site level PMTCT data to supplement or replace ANC sentinel surveillance and population survey HIV prevalence estimates. [20] While discrepancies in results across these methods need to be reconciled, use of site level PMTCT data is increasing to inform local program planning.

In 2012, the Mozambican Ministry of Health (*Ministério de Saúde*, [MISAU]), in conjunction with its U.S. PEPFAR partners, initiated a national “Acceleration Plan” for further expansion and scale-up of its HIV care and treatment programs in order to try and overcome its low ART coverage rates. This plan called for rapidly expanding services in Zambézia Province from the 31 health facilities offering ART in December 2012 to 104 by the end of 2014, though no additional financial resources were available given a plateauing of donor funding.

The rapid scale-up in numbers of persons taking ART to date, coupled with the continued demands for expansion to more health facilities in the midst of the current fiscal stagnation, underscores the need for prioritizing integrated health planning; and improved program evaluation that can rapidly inform evidence-based best practices. [3], [21]–[24] We examine the spatial patterns of community-based VCT delivery points with facility-based HIV program demographic information. We hypothesize that previously unrecognized geographic areas of high HIV concentration can be estimated, ultimately illuminating future priority areas of targeted community-based VCT campaigns, as well as provide the evidence needed to guide future ART expansion planning.

## Methods

### Ethical review

Both the Zambézia Province Inter-institutional Bioethics Committee for Health (*Comité Inter-institucional de Bioética para a Saúde da Provincia de Zambézia* [CIBS-Z]) and the Institutional Review Board of Vanderbilt University approved this analysis. Routine programmatic data stripped of identifiers were used for analysis. Since no participants were enrolled or interviewed, no informed consent was obtained.

### Study Context

In 2009, World Vision International was awarded the United States Agency for International Development (USAID) - Strengthening Communities through Integrated Programing (SCIP) grant, a 5-year multi-sector project aimed at improving the health and livelihoods of children, women and families in Zambézia Province. Known locally as *Ogumaniha*, which means “united for a common purpose” in the local language of Echuabo, *Ogumaniha* is a consortium of 5 organizations with a goal of integrating current and future US government investments in Zambézia Province in the areas of health, HIV/AIDS, water and sanitation, income generation, and institutional capacity-building.

Friends in Global Health (FGH), a Mozambican-registered NGO affiliated with the Vanderbilt Institute for Global Health (VIGH), spearheads monitoring and evaluation activities for the consortium. FGH is also a PEPFAR clinical partner providing technical assistance to the Zambézia Provincial Health Directorate (*Direcção Provincial da Saúde de Zambézia* [DPS-Z]) since 2007. *Ogumaniha* currently works in 16 of Zambézia’s 17 districts and FGH currently provides PEPFAR HIV care and treatment technical assistance to 10 districts, 9 of which overlap with *Ogumaniha* supported districts.

**Figure 1:**
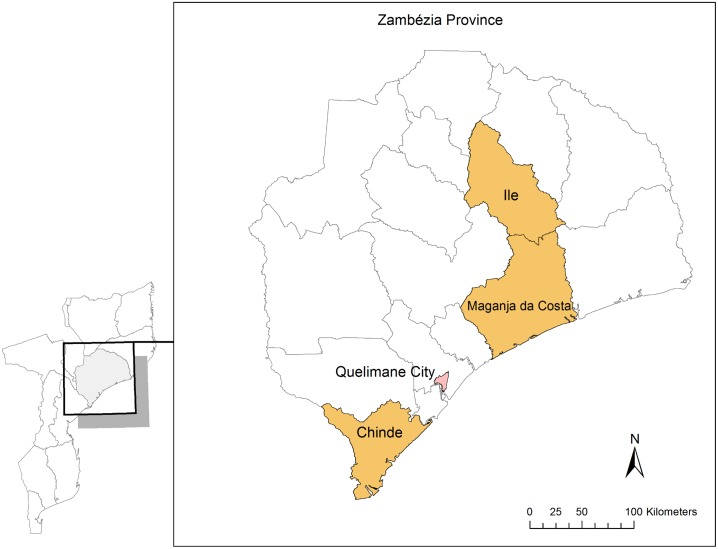
Map of Mozambique, Zambézia Province with Districts of Ile, Maganja da Costa, and Chinde Highlighted. The Provincial Capital of Quelimane and District of Inhassunge also Represented.

This analysis was conducted for the time period October 2011 to September 2012, in the rural districts of Ile, Maganja da Costa, and Chinde (Figure 1). These districts were chosen given that FGH staff work in all three through both the *Ogumaniha* project and as a PEPFAR clinical partner, and also because of the diversity of their HIV prevalence rates, both higher and lower than the 12.6% reported for the province as a whole [13].

### Data collection

We analyzed routine data collected from the *Ogumaniha* project on community-based VCT implementation and facility-based HIV care and treatment program data for map generation. Data were integrated within all maps. Data are available upon request to World Vision International and the National Bioethics Committee for Health, Maputo, Mozambique.

### Ogumaniha community-based VCT

The *Ogumaniha* community-based VCT program collects data using paper data collection tools developed by MISAU. Community-based VCT counselors conduct door-to-door counseling and testing; and when consent to undergo HIV testing is obtained the counselor manually completes a record on the paper data collection tool. At the end of the month, the counselor compiles the data into two different monthly summary forms: one that is submitted to the provincial and district health authorities and a second which is submitted for PEPFAR reporting requirements. Data are then entered by data entry staff into the *Ogumaniha* Information System (OgIS), an open source electronic data tracking system based on the District Health Information System II (DHIS II, www.dhis2.org). For this analysis we queried demographic information for persons who participated in community-based HIV counseling and testing and received their results between October 2011 and September 2012.

### Data collection for facility-based HIV care and treatment program

The facility-based HIV care and treatment program collects routine data as required by MISAU and for PEPFAR reporting requirements. [5], [6] Paper forms and registers are completed by clinicians, pharmacists, and lay counselors who meet with the patients. Data are entered after the patient visit by data entry staff at each site, into an Open Medical Record (OpenMRS) electronic patient tracking system maintained by the DPS-Z and FGH staff. [25] For this analysis we queried demographic information for newly enrolled patients between October 2011 and September 2012.

### Map generation

**Figure 2:**
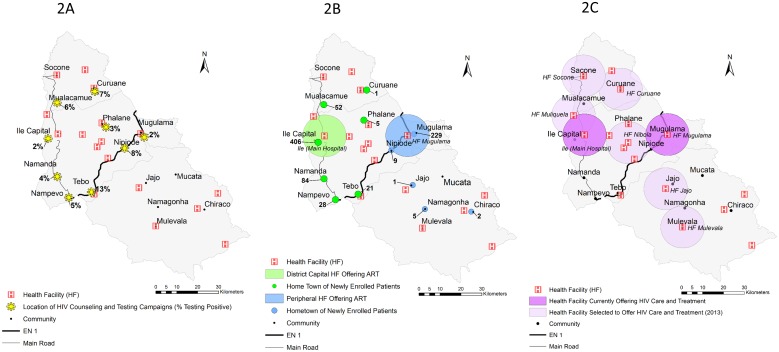
Map of Ile. *Figure 2A shows the geographic locations of communities that reported community-based VCT activities and the percent testing HIV positive, in the time period between October 2011 and September 2012. *Figures 2B shows the geographic locations of where patients live, that were newly enrolled into HIV care and treatment in the same time period between October 2011 and September 2012. The main hospital in the district capital providing ART services is marked with a 10 km radius (green) around the hospital, while the smaller peripheral health facility also currently providing ART services is marked in blue. Persons living in communities designated with a green dot enrolled in the ART site with 10 km radius in green. Persons living in communities designated with a blue dot enrolled in the ART site with a 10 km radius in blue. *Figures 2C shows the locations of health facilities in each district currently providing ART (purple) and those slated for expansion of HIV care and treatment services in 2013 (light purple).

**Figure 3:**
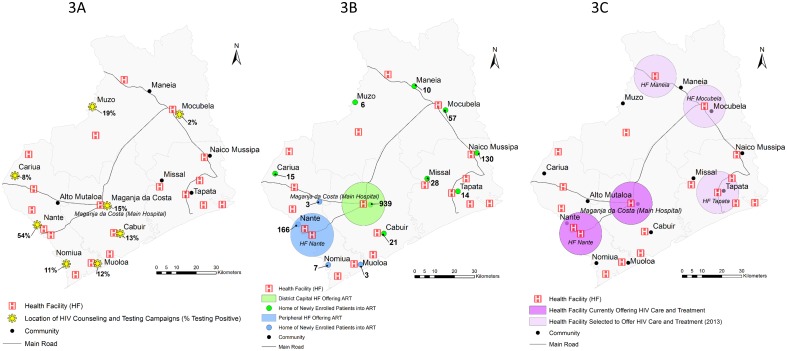
Map of Maganja da Costa. *Figure 3A shows the geographic locations of communities that reported community-based VCT activities and the percent testing HIV positive, in the time period between October 2011 and September 2012. *Figures 3B shows the geographic locations of where patients live, that were newly enrolled into HIV care and treatment in the same time period between October 2011 and September 2012. The main hospital in the district capital providing ART services is marked with a 10 km radius (green) around the hospital, while the smaller peripheral health facility also currently providing ART services is marked in blue. Persons living in communities designated with a green dot enrolled in the ART site with 10 km radius in green. Persons living in communities designated with a blue dot enrolled in the ART site with a 10 km radius in blue. *Figures 3C shows the locations of health facilities in each district currently providing ART (purple) and those slated for expansion of HIV care and treatment services in 2013 (light purple).

**Figure 4:**
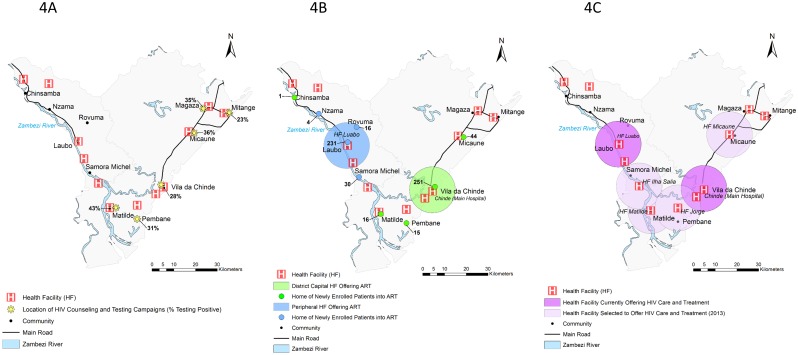
Map of Chinde. *Figure 4A shows the geographic locations of communities that reported community-based VCT activities and the percent testing HIV positive, in the time period between October 2011 and September 2012. *Figures 4B shows the geographic locations of where patients live, that were newly enrolled into HIV care and treatment in the same time period between October 2011 and September 2012. The main hospital in the district capital providing ART services is marked with a 10 km radius (green) around the hospital, while the smaller peripheral health facility also currently providing ART services is marked in blue. Persons living in communities designated with a green dot enrolled in the ART site with 10 km radius in green. Persons living in communities designated with a blue dot enrolled in the ART site with a 10 km radius in blue. *Figures 4C shows the locations of health facilities in each district currently providing ART (purple) and those slated for expansion of HIV care and treatment services in 2013 (light purple).

Each year *Ogumaniha* implements a province wide resource mapping exercise of all 214 DPS-Z health facilities and >800 community health committees (CHC’s) for which *Ogumaniha* provides support, using GPS-capacitated cell phones for in-field data collection. Data are temporarily stored in an open source database called Open Data Kit (ODK) Aggregate which subsequently translates into compatible data for final storage in REDCap (Research Electronic Data Capture) (http://www.project-redcap.org/). As a consequence, *Ogumaniha* has developed a significant repository of geographic coordinates (latitude/longitude), collected at health facilities and at the community center of communities in which CHC’s exist, from which we were able to cross reference community, health facility, and demographic information collected from the community-based VCT and facility-based HIV programs. A series of maps were then created using ArcGIS 10.1 depicting the locations of community-based VCT activities, home communities of those enrolled into HIV care and treatment, and locations of ART expansion for the three focus districts. District boundaries, administrative units, inland water bodies, and road data were obtained from DIVA-GIS Free Spatial Data (http://www.diva-gis.org/Data/). These data were originally based on data obtained from the Global Administrative Areas (GADM) (http://www.gadm.org/) and the Defense Mapping Agency (DMA) Digital Chart of the World, Fairfax, Virginia, 1992 (Figure 2–4).

## Results

There are 46 total health facilities in the three districts (17 in Ile, 19 in Maganja da Costa, and 10 in Chinde). At the end of September 2012, two health facilities in each district were offering ART services (6 total). Per the data reported by FGH in the PEPFAR Annual Progress Report (APR) for the period October 2011 to September 2012, ART coverage estimates (persons on ART as a proportion of estimated HIV positive persons eligible for ART denominator) were calculated to be 45% for Ile, 11% for Maganja da Costa, and 4% for Chinde (Table 1).

**Table 1:** ART Coverage Estimates: Zambézia Province and Three Focus Districts, Ile, Maganja da Costa, and Chinde.

	Pop. Est.2012	Est. # of HIVInfected^a^	Est. # Eligible forART^b^	# Active on ART(Sept 2012)^c^	ART Coverage^d^
Zambézia Province	3,800,000	478,800	134,064	23,453	17.5%
District of Ile	323,116	6,462	1,809	819	45%
District of Maganja da Costa	306,288	35,223	9,862	1,095	11%
District of Chinde	131,534	18,950	5,306	224	4%

a The Estimated Number of HIV Infected is calculated using the 2012 HIV prevalence estimates (Table 2), as a proportion of estimated population.

b The Estimated Number Eligible for ART is calculated by taking 28% of the Estimated Number of HIV Infected (Due to current lack of more detailed sub-regional data, 28% is the current Ministry of Health standard for calculating the estimated number eligible for ART. More detailed population-based surveys reaching the district and community level are planned to be conducted in late 2014).

c Number of persons active and on ART as reported in the PEPFAR Annual Progress Report (APR) for the period October 2011–September 2012.

d ART Coverage is defined as the Number Active on ART as of Sept 2012 (numerator) over the estimated number of HIV positive persons eligible for ART (denominator).

### Community-based VCT activities

Figures 2A, 3A, and 4A show the geographic locations of communities that reported community-based VCT activities in the time period between October 2011 and September 2012. In general, community-based VCT was implemented in areas with close geographic proximity to health facilities providing ART; within communities with easier transportation access; and/or near the homes of *Ogumaniha* VCT volunteers. Numbers of persons tested and number and percent of those testing HIV seropositive are in Table 2.

**Table 2:** Ogumaniha Community-Based Voluntary Counseling and Testing Campaigns: Ile, Maganja da Costa and Chinde Districts, October 2011–September 2012.

Location of Community-Based VCT	# Tested	# HIV positive	% HIV positive
**District of Ile**	**-**	**-**	**-**
Ile (main health facility)	1,044	16	2%
Namanda	628	28	4%
Mugulama	155	3	2%
Phalane	123	4	3%
Nampevo	365	18	5%
Nipiode	50	4	8%
Mualacamue	85	5	6%
Curuane	30	2	7%
Tebo	30	4	13%
Total from Community-Based VCT	2,510	84	**3%**
2012 District HIV Prevalence Est*	-	-	**2%**
**District of Maganja da Costa**	**-**	**-**	**-**
Maganja da Costa (main health facility)	1,732	258	15%
Cabuir	124	16	13%
Cariua	484	37	8%
Mocubela	171	4	2%
Muzo	16	3	19%
Muolo	17	2	12%
Nante	462	248	54%
Nomiua	54	54	11%
Total from Community-Based VCT	3,060	622	**20%**
2012 District HIV Prevalence Est*	-	-	**11.5%**
**District of Chinde**	-	**-**	**-**
Vila de Chinde (main health facility)	623	173	28%
Pambane	35	11	31%
Matilde	30	13	43%
Micaune	452	162	36%
Magaza	34	12	35%
Mitange	40	9	23%
Total from Community-Based VCT	1,214	380	**31%**
2012 District HIV Prevalence Est*	-	-	**14.4%**
2012 Zambézia HIV Prevalence Est**	-	-	**12.6%**

*District Prevalence Estimates are based on the percent of pregnant women testing HIV seropositive at antenatal care clinics (routine PMTCT data) from the districts main health facility for the period October 2011–September 2012.

**Zambézia Province HIV prevalence as reported by INSIDA, 2009^13^.

### Location of persons newly enrolled into HIV care and treatment

Figures 2B, 3B, and 4B show the geographic locations of communities where patients live who were newly enrolled into HIV care and treatment between October 2011 and September 2012, juxtaposing their locations to those of the health facilities providing ART in which they were enrolled. Health facilities providing ART services are marked with a 10 km radius. While we know persons outside the 10 km radius do travel in to the health facility, travel distance by foot within this 10 km radius is generally considered a tolerable distance in Mozambique for purposes of adherence and retention planning.

### District of Ile

In the district of Ile (Figure 2A) community-based VCT activities were concentrated heavily in the district’s northwest, in areas closest to the national highway and in transport corridors leading to significant commercial centers in surrounding districts. In these areas of higher VCT coverage, the proportions of persons testing HIV seropositive (2–13%) through the community-based VCT activities of *Ogumaniha* trended higher than the prevalence estimates calculated from routine PMTCT data from the main district health facility (2%). The home locations of newly enrolled patients into HIV care and treatment in Ile (Figure 2B) closely mirrored those areas in which community-based VCT activities were implemented, with very few newly enrolled patients coming from the southeastern part of the district in which no community-based VCT activities took place.

### District of Maganja da Costa

In the district of Maganja da Costa (Figure 3A), community-based VCT activities were again heavily concentrated geographically, this time in the district’s west, in areas of easiest transportation access. Maganja da Costa reports a much wider range than Ile in the proportion of persons testing HIV seropositive in different community-based VCT venues (2%–54%). Community-based VCT in the capital city of Maganja da Costa (site of the main district health facility) reported HIV positivity to be 15% of those tested, which is relatively consistent with the PMTCT testing (Table 2) used to estimate the district’s prevalence. However, the 11.5% prevalence estimated through routine PMTCT testing likely does not represent the district as a whole, given pockets of much higher (54% in Nante) and lower (2% in Mocubela) seroprevalence.

The eastern region of Maganja da Costa is more isolated with extremely long travel times between communities. For example, to travel by car between Maganja da Costa (the capital city) and Tapata, one must first travel approximately 2½ hours north to Mocubela followed by an additional 1½ hour south to Tapata. Despite significant travel distances, newly enrolled patients into HIV care and treatment during the reporting period come from fairly dispersed communities across the district (Figure 3B). About 20% of patients newly enrolled in the reporting period came from areas requiring more than a 2 hour drive to reach HIV care and treatment services. The large numbers of newly enrolled patients coming from Naico Mussipa, Mocubela, and Tapata/Missal, despite having no organized community-based VCT activities occurring during the reporting period, could suggest that the eastern region of the district represents a previously unrecognized region of unmet need.

### District of Chinde

The district of Chinde is geographically isolated and is uniquely difficult to work in compared to other provincial districts. Extremely limited in transportation options, the district is divided in two by the Zambezi River which runs along the western border, cutting across the district and connecting Luabo and the district capital of Vila de Chinde. In order to reach the district capital from Quelimane (the provincial capital), one must drive 6 hours from Quelimane to Luabo and then catch a boat from Luabo to Vila de Chinde. The trip varies from 2 hours by a rapid private boat to 12 hours by public boat. Due to the fact that the only transport connection between Luabo and Vila de Chinde is by river, Luabo and its surrounding communities function relatively independent from the main administrative center of the district. Very poor roads connect Vila de Chinde up to the northeastern towns of Micaune, Mitange, and Magaza. During the rainy season, motorcycles are often the only viable form of transportation between these areas. Frequently it is easier to reach the towns in the northeast by crossing the border from Inhassunge district to the north rather than from within the district itself (Figure 1). As a result of logistic difficulties, community-based VCT activities are focused heavily in the geographic areas close to the district capital and sites easily reached from neighboring Inhassunge district.

The Chinde district’s HIV prevalence of 14.4%, as estimated through routine PMTCT testing, may represent an underestimation of its true prevalence, since the proportions of persons testing HIV-positive in community-based VCT activities was consistently higher than the antenatal clinic estimates (Figure 4A). Newly enrolled patients into HIV care and treatment at the Vila de Chinde health facility largely come from communities where community-based VCT took place (Figure 4B). In the reporting period, no community-based VCT activities occurred in the communities that feed into the Luabo health facility (Figure 4B).

### Acceleration plan for ART expansion

Figures 2C, 3C, and 4C show the locations of health facilities in each district currently providing ART (purple) and those that were selected for expansion of ART services (light purple) in 2013 as part of the Provincial Acceleration Plan. The acceleration plan consists of the target number of new ART facilities provided from MISAU at the national level, with implementation to be determined at the provincial level. Provincial planning activities between provincial health planners and partner organizations included presentation of both the community VCT and health facility data presented here. Selection of expansion sites was determined based on these results plus budgetary considerations for FY 2013. Each ART site is buffered with a 10 km radius to better illustrate the surrounding catchment area generally felt to be close enough for walking to receive care.

## Discussion

The use of geospatial mapping allowed for the intersecting of data from two different HIV programs and represents an effective example of joint program planning and monitoring as part of MISAU and US Government efforts to strengthen linkages between its community- and clinic-based HIV funded activities. This is the first such exercise in Zambézia Province in which routine program data were integrated with available geographic data, allowing for visualization of community-based VCT delivery points with the known locations of our HIV-infected constituents at the district level. This use of routine program data attempts to complement the existing national and provincial level HIV prevalence data, with district and site level prevalence estimates. While we recognize that comparisons of HIV prevalence between VCT and antenatal clinics is not ideal, due to large potential characteristic differences between these two populations and due to the fact that VCT often over-estimates HIV prevalence by selecting higher-risk individuals, accurate population density data and survey capacity in Mozambique does not currently exist for a more refined estimate of prevalence at the community level. By attempting to fill in the existing knowledge gap in district and site level data with estimates from the data we do have, program implementers were able to identify geographic areas of high HIV concentration, characterizing the location and size of each district’s HIV population and thus make evidence-based decisions of where to target future community-based VCT campaigns which will best support ART program expansion.

The finding that most community-based VCT activities in each of the three districts were concentrated within distinct geographic areas is consistent with the all too common inequity and uneven distribution of resources seen in Zambézia Province. Better served areas tend to have better infrastructures and ease of access. Despite the fact that community-based VCT activities have identified large numbers of HIV-positive persons in these districts and facility-based programs have enrolled large numbers of persons into HIV care and treatment, limitations to the analysis of community-based VCT data do exist. For example, to date the VCT and care/treatment programs have been working in relative isolation, with poor coordination between their respective planning efforts. Additionally, since community-based VCT is anonymous in Mozambique, minimal data describing the patients who tested positive during community VCT is currently collected, thus limiting our ability to analyze if these persons sought and enrolled in services, what the predictors of care uptake are, and whether or not the same persons have been HIV tested before/elsewhere. In Zambézia Province, patients tend to enroll in HIV care and treatment only after they have become sick and are symptomatic. [6] This suggests that in those communities reporting new enrollees and in which community-based VCT activities did not occur, the numbers of known HIV positive persons (as reflected by the number of new enrollees) is an under-representation of the true prevalence in those communities, representing previously unrecognized areas of need.

To increase ART coverage, programs must increase the number of persons aware of their HIV status and then ensure that those same persons have appropriate linkage and access to needed services. During its annual strategic planning meetings for the October 2013 to September 2014 year, *Ogumaniha* program implementers evaluated these data with provincial health authorities and with HIV care and treatment partner organizations, such as FGH. Lessons learned were discussed (Table 3) and the following concrete actions were established to be implemented in the upcoming programmatic year.

1) Community-based VCT efforts implemented by *Ogumaniha* will be focused on identified geographic areas of previously unmet need, that are in close proximity to the health facilities that will offer HIV care and treatment services under the Provincial ART Acceleration Plan.
2) Facility-based HIV care and treatment technical assistance provided by FGH will improve systems at the health facilities to receive, document, and follow those HIV positive persons referred through *Ogumaniha*’s community-based VCT activities. This will result in improved ability to document if persons testing positive have enrolled in HIV care and their subsequent progression through the cascade of events eventually leading to treatment. Linking persons directly from community-based testing to the HIV care and treatment records will additionally provide for more in-depth understanding of patient characteristics allowing for analyses such as examining predictors of HIV care and treatment uptake.
3) Geospatial mapping will be further incorporated into program planning and monitoring. Replication of this analysis in the remaining 14 districts of Zambézia Province has been requested by both Provincial Health Authorities and US government agencies and is underway.
4) Efforts are ongoing to define future studies to aid in understanding the marked inter-district and intra-district geographic variations often seen in HIV prevalence.

Current ART expansion roll-out as defined in Zambézia’s Provincial Acceleration Plan and as shown in figures 2C, 3C, and 4C should provide a sufficient geographic dispersion of health facilities providing ART to dramatically increase coverage of these districts. Based on the available information presented here, as ART expansion continues beyond the current planning, sites of potential unmet need to be considered for future ART expansion would include: Tebo and Chiraco in the district of Ile; Naico Mussipa, Muoloa, and Cariua in the district of Maganja da Costa; and Chinsamba, Magaza and Mitange in the district of Chinde.

As HIV care and treatment programs continue to evolve, striving for greater access and quality in service delivery, efforts to “*know your epidemic*” at the local level will require better micro-planning at district and community levels. Integrating a framework for geospatial mapping can identify areas of need and help planners set priorities for service investments that maximize the benefits to the population.

## References

[1] El-Sadr WM, Holmes CB, Mugyenyi P, Thirumurthy H, Ellerbrock T, et al (2012) Scale-up of HIV treatment through PEPFAR: a historic public health achievement. J Acquir Immune Defic Syndr 60 Suppl 3S96–104. 22797746 10.1097/QAI.0b013e31825eb27b PMC3445041

[2] Lahuerta M, Ue F, Hoffman S, Elul B, Kulkarni SG, et al (2013) The problem of late ART initiation in Sub-Saharan Africa: a transient aspect of scale-up or a long-term phenomenon? J. Health Care Poor Underserved 24(1): 359–383. 23377739 10.1353/hpu.2013.0014 PMC3655523

[3] De Cock KM, El-Sadr WM, Ghebreyesus TA (2011) Game changers: why did the scale-up of HIV treatment work despite weak health systems? J Acquir Immune Defic Syndr 57 Suppl 2S61–63. 21857297 10.1097/QAI.0b013e3182217f00

[4] Vermund SH, Sidat M, Weil LF, Tique JA, Moon TD, et al (2012) Transitioning HIV care and treatment programs in southern Africa to full local management. AIDS 26(10): 1303–1310. 22706012 10.1097/QAD.0b013e3283552185 PMC3576840

[5] Moon TD, Burlison JR, Sidat M, Pires P, Silva W, et al (2010) Lessons learned while implementing an HIV/AIDS care and treatment program in rural Mozambique. Retrovirology: Research and Treatment 3: 1–14. 25097450 10.4137/RRT.S4613 PMC4119752

[6] Moon TD, Burlison JR, Blevins M, Shepherd BE, Baptista A, et al (2011) Enrolment and programmatic trends and predictors of antiretroviral therapy initiation from president’s emergency plan for AIDS Relief (PEPFAR)-supported public HIV care and treatment sites in rural Mozambique. Int J STD AIDS 22(11): 621–627. 22096045 10.1258/ijsa.2011.010442

[7] Groh K, Audet CM, Baptista A, Sidat M, Vergara A, et al (2011) Barriers to antiretroviral therapy adherence in rural Mozambique. BMC Public Health 11: 650. 21846344 10.1186/1471-2458-11-650 PMC3171727

[8] Ciampa PJ, Skinner SL, Patricio SR, Rothman RL, Vermund SH, et al (2012) Comprehensive knowledge of HIV among women in rural Mozambique: development and validation of the HIV knowledge 27 scale. PLOS ONE 7(10): e48676. 23119087 10.1371/journal.pone.0048676 PMC3485372

[9] Audet CM, Sidat M, Blevins M, Moon TD, Vergara A, et al (2012) HIV knowledge and health-seeking behavior in Zambézia Province, Mozambique. SAHARA J: J Soc Aspects HIV/AIDS Res Alliance 9(1): 41–46. 10.1080/17290376.2012.665257 PMC3868210 23237020

[10] Audet CM, Burlison J, Moon TD, Sidat M, Vergara A, et al (2010) Sociocultural and epidemiological aspects of HIV/AIDS in Mozambique. BMC Int Heal Hum Rights 10: 15. 10.1186/1472-698X-10-15 PMC2891693 20529358

[11] Focus Countries. The President’s Emergency Plan for AIDS Relief (PEPFAR). Available: http://www.pepfar.gov/. Accessed 24 Sept 2014.

[12] International Human Development Indicators - UNDP. Available: http://hdr.undp.org/en/countries/profiles/MOZ. Accessed 24 Sept 2014.

[13] INSIDA 2009, Inquérito Nacional de Prevalência, Riscos Comportamentais e Informação sobre o HIV e SIDA em Moçambique. Available: http://dhsprogram.com/pubs/pdf/AIS8/AIS8.pdf. Accessed 24 Sept 2014.

[14] Moçambique Inquérito Demográfico e de Saúde 2011-Demographic Health Survey 2011. Available: http://www.measuredhs.com/pubs/pdf/FR266/FR266.pdf. Accessed 24 Sept 2014.

[15] MICS Summary English - Multiple Indicator Cluster Survey (2008) Available: http://www.unicef.org/mozambique/MICS_Summary_English_201009.pdf. Accessed 24 Sept 2014.

[16] NCMS Summary English-National Child Mortality Survey (2009) Available: http://www.unicef.org/mozambique/NCMS_Summary_Report_ENG_220909.pdf. Accessed 24 Sept 2014.

[17] Cook RE, Ciampa PJ, Sidat M, Blevins M, Burlison J, et al (2011) Predictors of successful early infant diagnosis of HIV in a rural district hospital in Zambézia, Mozambique. J Acquir Immune Defic Syndr 56(4): e104–109. 21266912 10.1097/QAI.0b013e318207a535 PMC3073723

[18] Ciampa PJ, Vaz LME, Blevins M, Sidat M, Rothman RL, et al (2012) The association among literacy, numeracy, HIV knowledge and health-seeking behavior: a population-based survey of women in rural Mozambique. PLOS ONE 7(6): e39391. 22745747 10.1371/journal.pone.0039391 PMC3382184

[19] HIV/SIDA. Ministério da Saúde-Moçambique. Available: http://www.misau.gov.mz/index.php/hiv-sida. Accessed 24 Sept 2014.

[20] Young PW, Mahomed M, Horth RZ, Shiraishi RW, Jani IV, et al (2013) Routine data from prevention of mother-to-child transmission (PMTCT) HIV testing not yet ready for HIV surveillance in Mozambique: a retrospective analysis of matched test results. BMC Infect Dis 13: 96. 23432847 10.1186/1471-2334-13-96 PMC3598230

[21] Larson E, O’Bra H, Brown J, Mbengashe T, Klausner JD (2012) Supporting the massive scale-up of antiretroviral therapy: the evolution of PEPFAR-supported treatment facilities in South Africa, 2005–2009. BMC Public Health 12: 173. 22404862 10.1186/1471-2458-12-173 PMC3323417

[22] Boyer S, Koulla-Shiro S, Abé C, Spire B, Moatti JP (2011) Implementing operational research to scale-up access to antiretroviral therapy for HIV infection: lessons learned from the Cameroonian experience. Curr Opin HIV AIDS 6(4): 239–244. 21537170 10.1097/COH.0b013e3283478757

[23] Harries AD, Makombe SD, Libamba E, Schouten EJ (2011) Why Did the Scale-up of HIV Treatment Work?: A Case Example From Malawi. J Acquir Immune Defic Syndr 57: S64–S67. 21857298 10.1097/QAI.0b013e31821f6bab

[24] Ciampa PJ, Tique JA, Jumá N, Sidat M, Moon TD, et al (2012) Addressing poor retention of infants exposed to HIV: a quality improvement study in rural Mozambique. J Acquir Immune Defic Syndr 60(2): e46–52. 22622077 10.1097/QAI.0b013e31824c0267 PMC3587032

[25] Manders EJ, José E, Solis M, Burlison J, Nhampossa JL, et al (2010) Implementing OpenMRS for patient monitoring in an HIV/AIDS care and treatment program in rural Mozambique. Stud Heal Technol Inform 160: 411–415. 20841719

